# Longitudinal study of pulmonary function trends and associated risk factors in iron ore miners

**DOI:** 10.1038/s41598-025-19091-7

**Published:** 2025-09-29

**Authors:** Haniyeh Soltanpour, Ali Faghihi Zarandi, Abdollah Gholami, Saiedeh Haji-Maghsoudi, Behnam Khodarahmi, Pejman Mohammadi, Rouhollah Parvari

**Affiliations:** 1 Department of Occupational Health Engineering and Safety at Work, Faculty of Public Health, Kerman University of Medical Sciences, Kerman, 7616913555 Iran; 2 Department of Occupational Health, School of Health, Social Department of Health Research Center, Birjand University of Medical Sciences, Birjand, 9717853076 Iran; 3 Modeling in Health Research Center, Institute for Futures Studies in Health, Kerman University of Medical Sciences, Kerman, 7616913555 Iran; 4 Department of Occupational Health Engineering, Gol-E-Gohar Mining and Industrial Co, Sirjan, 7616913555 Iran; 5 Department of Occupational Health and Safety Engineering, School of Health, Ardabil University of Medical Sciences, Ardabil, 5618953141 Iran; 6 Environmental Health Engineering Research Center, Kerman University of Medical Sciences, Kerman, 7616913555 Iran

**Keywords:** Longitudinal changes, Pulmonary functions, Miners, Iron dust, Risk factors, Silica., Occupational health, Respiratory signs and symptoms, Risk factors

## Abstract

The mining industry involves various processes, including extraction, crushing, milling, stacking, and reclaiming mineral substances. Workers in these environments are at risk of respiratory diseases due to exposure to high concentrations of pollutants, such as respirable dust. This study was conducted on sweepers, supervisors, and office workers of an iron ore Concentrate and Raw pellet production plants. Sampling and analysis of respirable dust, crystalline silica, and iron dust were performed according to NIOSH 0600, NIOSH 7601, and OSHA ID-121 methods, respectively. The values ​​of lung function indices were extracted from personnel medical records over the years of their work experiences. The results showed that the highest mean concentration of respirable dust, iron, and crystalline silica dust belongs to the sweeper group and the lowest mean concentration belongs to the office group. The reduction rate of pulmonary functions over time was also higher in the sweeper and supervisor groups than in the office group. The effective factors on the pulmonary functions were age, work experience, academic education level, occupational group, cigarette and waterpipe smoking, and type of employment.

**Supplementary Information:**

The online version contains supplementary material available at 10.1038/s41598-025-19091-7.

## Introduction

The mining industry involves various processes, including the extraction, crushing, milling, stacking, and reclaiming of mineral substances. Workers in these environments are often exposed to high concentrations of pollutants, such as respirable dust. These exposures can lead to respiratory diseases, ranging from common coughs to lung cancer. The differences in the ingredients of mineral ores, as well as variations in mining techniques and methodologies across different mines, may be one reason for the difference in the health problems reported in numerous studies within this field^1,2^.

In occupational health, risk is defined as the probability of adverse health effects or harm resulting from exposure to workplace hazards^2^. Miners are exposed to numerous occupational health risks, influenced by factors like the composition and size of dust particles in their environment^3^. Iron and crystalline silica are the primary pollutants in the iron ore mines^4^.

Iron is a necessary element for several essential body functions, such as enzyme activity. However, if the iron value in the body exceeds a certain level, it can lead to diseases such as diabetes, siderosis (a type of pneumoconiosis), and liver cancer. Additionally, dusts from mining processes often contain crystalline silica that can result in respiratory diseases such as silicosis, lung cancer, chronic pulmonary diseases, and autoimmune disorders when inhaled in high concentrations over long periods.

Crystalline silica, unlike its amorphous form, is insoluble in body fluids and demonstrates a tendency to accumulate within the lungs^5^. Respirable silica particles, typically less than 5 microns in size, can reach the most distal regions of the lungs, where they tend to accumulate. To eliminate these particles, the body’s macrophages migrate to these areas and engulf them. Over time, pulmonary nodules may develop at these sites, which are composed of macrophages that have ingested silica particles. Such nodules can lead to respiratory problems^6^.

Considering that millions of workers worldwide are faced with these conditions, investigation and research in this field have a high priority^1,4^.The National Institute for Occupational Safety and Health (NIOSH) estimates that each year, a large number of people who are exposed to these pollutants suffer from respiratory diseases and some even lose their lives^7^. Studies also indicate that occupational exposure to dust in the workplace is associated with an increased prevalence of chronic obstructive pulmonary disease (COPD), development of both acute and chronic respiratory problems, and kidney, liver, and other respiratory diseases^1,8,9^.

In a longitudinal study of 871 iron ore miners, it was found that individuals with moderate protein deficiency had a significantly greater decline in the FEV₁/FVC ratio. These findings highlight the impact of occupational and genetic conditions on pulmonary function^10^.

With the increase in population and the resulting increased need for iron metal, the number of workers exposed to high levels of mining dust is rising. This situation calls for greater attention to monitoring these workers’ health and addressing the associated risks^9^.

Sweepers are individuals who clean work surfaces and floors, either manually or using machines. Several studies have been conducted on street sweepers^11–18^; however, industrial sweepers, particularly those working in mines, face challenging conditions. To our knowledge, no dedicated studies have been conducted on these workers to date. Another job group in mines is supervisors. This category included individuals involved in overseeing technical operations and conducting routine maintenance and repairs along the production lines. While some members held engineering roles, others were skilled technicians or line operators. Third group are Office workers, comprised of administrative personnel such as accountants, clerks, and secretaries, who are stationed in office environments within the mine premises.

This study was conducted in an open-pit iron ore mining complex that include Concentration and Pelletizing (Raw Pellet production) plants. In the Concentrate production plant, extracted ore is crushed, ground, and processed through magnetic separation to produce iron ore concentrate. The concentrate is then processed in the Pelletizing unit, where it is mixed with binders, shaped into green pellets, and heat-treated to produce hardened pellets. These operations involve multiple stages of material handling and high dust-generating activities, potentially leading to elevated airborne particulate levels in the workplace^19,20^. In this study, we aimed to assess the occupational exposure to respirable dust, crystalline silica, and iron dust in three groups of personnel including sweepers, supervisors and office workers (as control group) and to track longitudinal changes in pulmonary functions to identify the factors affecting them in an open-pit iron ore mining complex in Iran.

## Materials and methods

This study, carried out from 2022 to 2023 at an iron ore mining complex in Iran—which included Concentration and Pelletizing plants—aimed to assess longitudinal changes in pulmonary function and associated risk factors among miners. All participants were male. The inclusion criteria were as follows: employment starting in 2018 or earlier, and no pre-existing lung or cardiovascular diseases at the time of employment. One objective of this study was to investigate changes in pulmonary function parameters; to objective this, it was necessary to include only workers with spirometry records for two consecutive years.

Based on the study’s purpose and design, three occupational groups were included: Group 1 consisted of 120 sweepers, who spent extended periods in areas with high dust concentrations; Group 2 included 120 supervisors, who spent less time in these high-dust areas than the sweepers; and Group 3 comprised 120 office personnel. All participant completed and signed informed consent form to participate in the study and were given the freedom to participate or withdraw from the study at any stage of the research. The research protocol was approved by the university ethics committee (Ethical code: IR.KMU.REC.1402.396) and performed in accordance with the Helsinki Declaration of 1964 as revised in 2000.

Demographic data was collected using a checklist. This research was done in the following steps: First, personnel exposures to pollutants (Respirable dust, Crystalline silica, and Iron dust) were determined. In this relation, Sampling and analysis of respirable dust, crystalline silica, and iron dust were done according to NIOSH 0600, NIOSH 7601, and OSHA ID-121 methods, respectively^21^.

Crystalline silica sampling and analysis were conducted following NIOSH Method 7601. A plastic cyclone equipped with a PVC filter was used for silica sampling with an air flow rate maintained at 3 L/min. Preliminary tests were performed to determine the optimal sampling duration for each sample. Sample preparation involved treatment with nitric acid, perchloric acid, phosphoric acid, hydrofluoric acid, boric acid, molybdate reagent, and sulfuric acid. Analysis was performed using a UV/VIS spectrophotometer, with absorbance measurements taken at either 420–820 nm. A calibration curve was generated using Excel software, and the absorption-concentration equation was established through linear regression model (Figure S1). The crystalline silica concentration (C, mg/m³) was subsequently calculated using Eq. 1.

1 $$\:C=\frac{(A-B)}{m\:\times\:V}$$

Where *A* and *B* represent the absorbance of the sample and blank, respectively; *m* denotes the slope of the calibration curve (µg^− 1^) and *V* is the sampled air volume (liter).

Respirable dust sampling and analysis were performed according to the NIOSH-0600 method. The sampling procedure followed the same protocol as described for crystalline silica dust. For analysis, filters were weighed before and after sampling using a high-precision analytical balance (RADWAG AS-R220/60, sensitivity: 0.00001 g). The respirable dust concentration (*C*, mg/m³) was then calculated using Eq. (2):

2 $$\:\text{C}=\frac{\left({\text{W}}_{2}-{\text{W}}_{1}\right)-({\text{B}}_{2}-{\text{B}}_{1})}{\text{V}}\times\:{10}^{3}$$

Where *W*_*1*_ and *W*_*2*_ are the filter weights before and after sampling (mg), respectively; *B*_*1*_ and *B*_*2*_ denote the corresponding blank filter weights before and after sampling (mg), and *V* is the total sampled air volume (liter).

Iron dust concentrations were measured following the OSHA ID-121 standard method. Sampling procedures were identical to those used for crystalline silica, except that mixed cellulose ester (MCE) filters were substituted for PVC filters. Prior to analysis, samples underwent acid digestion using two distinct solutions for ashing and dilution. Quantitative analysis was performed using a Varian AA240 atomic absorption spectrometer equipped with a flame (acetylene/air) atomizer, with measurements taken at the iron-specific wavelength of 248.3 nm. A calibration curve was generated using iron standard solutions, from which an absorption-concentration equation was established (Figure S2). The iron dust concentration (C, mg/m³) was then calculated using Eq. (3):

3 $$\:\text{C}=\frac{\left({\text{C}}_{\text{s}}{\text{V}}_{\text{s}}\right)-\left({\text{C}}_{\text{b}}{\text{V}}_{\text{b}}\right)}{\text{V}}$$

Where *C*_*s*_ and *C*_*b*_ represent the solution concentration of the sample and blank (µg/ml), respectively; *V*_*s*_ and *V*_*b*_ denote the corresponding solution volumes (ml); and *V* is the total sampled air volume (liter).

To ensure analytical repeatability, all sample analyses were performed under controlled conditions: the same methodology, operator, instrumentation, environmental conditions, and laboratory location were maintained for each pollutant type. The high coefficient of determination (R² > 0.98) obtained from the calibration curves (Figures S1 and S2) further confirmed the good repeatability of the analytical procedures.

The required number of air samples was determined using the similar exposure group (SEG) method. The 360 participants were classified into SEGs based on their work conditions, job, and tasks. As a result, 120 sweepers and 120 supervisors were each divided into 7 SEGs, while all 120 office workers fell into a single SEG due to their similar working conditions. SEGs were determined through observations of work processes and exposure conditions. Workers with similar exposure profile were grouped into appropriate SEGs^22^ and 73 air samples were taken for each pollutant. Accordingly, air sampling was conducted on 31 sweepers, 34 supervisors, and 8 office workers—representing their respective SEGs.

The next step of the research was to collect the pulmonary function values, which were extracted from the annual medical records of personnel. The collected pulmonary function indices were FVC, FEF, FEV_1_, PEF, and the FEV_1_/FVC. After extracting the data from medical records, their longitudinal changes and the factors affecting them were analyzed using related statistical tests.

Descriptive statistics, including the mean and standard deviation (SD), as well as the median with interquartile ranges (first and third quartiles), were calculated for the variables of interest. Frequencies and percentages were reported for categorical variables. The normality of the data was assessed using the Shapiro-Wilk test. When the normality assumption was rejected, the Kruskal-wallis test was employed to evaluate differences across occupational groups. If the Kruskal-wallis test indicated statistical significance, Dunn’s test was applied for pairwise comparisons, with p-values adjusted using the Bonferroni correction. Associations between categorical variables were examined using the Chi-square test. Linear mixed models were utilized to evaluate trends and factors associated with pulmonary function. For each response variable, two models were developed. Model 1 included occupational group, work experience, and their interaction. Model 2 was built upon Model 1 by adjusting for confounding variables. A significance level of 0.05 was used for all analyses. All statistical analyses were conducted using STATA version 17, and graphs were created in R software version 4.1.3.

## Results

### Demographic characteristics

Demographic characteristics of the studied personnel are provided in Tables 1 and 2. The variables of age, body mass index, and work experience among the three occupational groups didn’t have a statistically significant difference (P-value _age_ = 0.450, P-value _BMI_ = 0.940, and P-value _work experience_ = 0.798).

**Table 1:** Distribution of basic and demographic quantitative characteristics.

Variables	Occupational group			
	Sweeper	Supervisor	Office workers	*P*-value
	Mean ± SD	Mean ± SD	Mean ± SD	
Age (year)	37.61 ± 8.70	37.66 ± 5.91	37.40 ± 3.71	0.450^*^
BMI (kg/m^2^)	25.23 ± 3.12	25.34 ± 3.22	25.33 ± 2.40	0.940^*^
Work experience (year)	5.02 ± 1.08	5.09 ± 1.07	5.09 ± 1.06	0.798*

*Kruskal-Wallis test for comparing quantitative variables.

**Table 2:** Distribution of basic and demographic qualitative characteristics.

Variables		Occupational group			P-value
		Sweeper	Supervisor	Office workers	
		Number (Percentage)	Number (Percentage)	Number (Percentage)	
Work schedule number (%)	Day work	120 (100.00)	0 (0.00)	120 (100.00)	< 0.001^**^
	Shift Work	0 (0.00)	120 (100.00)	0 (0.00)	
Employment status number (%)	Corporate	120 (100.00)	60 (50.00)	45 (37.50)	< 0.001^**^
	Contractual	0 (0.00)	40 (33.30)	39 (32.50)	
	Official	0 (0.00)	20 (16.60)	36 (30.00)	
Marriage number (%)	Single	30 (25.00)	55 (45.80)	64 (53.30)	< 0.001^**^
	Married	90 (75.00)	65 (54.10)	56 (46.60)	
Education level number (%)	Under diploma	38 (31.60)	5 (4.10)	1 (0.80)	< 0.001^**^
	Diploma	40 (33.30)	15 (12.50)	10 (8.30)	
	Bachelor	39 (32.50)	80 (66.60)	88 (73.30)	
	Masters and above	3 (2.50)	10 (8.30)	21 (17.50)	
Smoking number (%)	Cigarette	9 (7.50)	70 (58.30)	60 (50.00)	< 0.001^**^
	Waterpipe	100 (83.30)	101 (84.10)	102 (85.00)	0.939

**Chi-square test for comparing qualitative variables.

The qualitative variables of the study are listed in Table 2. These variables were significantly different in the three studied groups (P-value < 0.001). Chi-square test was conducted to examine smoking behavior among the three studied occupational groups, and it was determined that there was a significant difference between the groups in terms of cigarette smoking (P-value < 0.001), but there was no significant difference for waterpipe consumption (P-value = 0.939).

### Personal exposure to pollutants

Crystalline silica dust and respirable dust exposure were higher than the threshold limit values (TLVs; 0.025 mg/m^3^ and 3 mg/m^3^, respectively) in sweepers and supervisors and lower than the threshold limit in the office group. The iron dust exposure level was lower than the threshold limit (5 mg/m^3^) in all three occupational groups.

The mean exposure concentration of crystalline silica, iron dust, and respirable dust among the three occupational groups had a statistically significant difference (P-value < 0.001), so that in sweepers it was higher than supervisors and in supervisors it was higher than office group (Table 3). Based on the Dun statistical test, the concentrations of crystalline silica and iron dusts showed statistically significant differences between the study groups when compared in pairs (P-value < 0.001).

**Table 3:** Comparison of pollutant exposure in three studied occupational groups.

Measured pollutant	Occupational group						
	Sweeper		Supervisor		Office workers		*P*-value
	Sample number	Mean ± SD	Sample number	Mean ± SD	Sample number	Mean ± SD	
Respirable dust	31	19.80 ± 20.80	34	3.97 ± 3.22	8	0.34 ± 0.12	< 0.001
Crystalline silica dust	31	0.62 ± 1.41	34	0.05 ± 0.04	8	0.002 ± 0.001	< 0.001
Iron dust	31	3.11 ± 1.91	34	1.09 ± 0.60	8	0.05 ± 0.01	< 0.001

Table 4 compares the studied pollutant exposures with the TLVs. For all three pollutants, in the sweeper group, the number of persons with exposure higher than threshold limits is greater than the other two groups.

**Table 4:** Comparison of exposure levels with tlvs.

Occupational group	Pollutant	Sample number	Employees with exposure higher than the TLV*	
			Number	Percentage
Sweeper	Respirable dust	31	27	87.10
	Crystalline silica dust	31	29	93.50
	Iron dust	31	6	19.30
Supervisor	Respirable dust	34	16	43.20
	Crystalline silica dust	34	25	67.50
	Iron dust	34	0	0.00
Office workers	Respirable dust	8	0	0.00
	Crystalline silica dust	8	0	0.00
	Iron dust	8	0	0.00

*TLV, Threshold limit values.

### Pulmonary functions

**Fig. 1:**
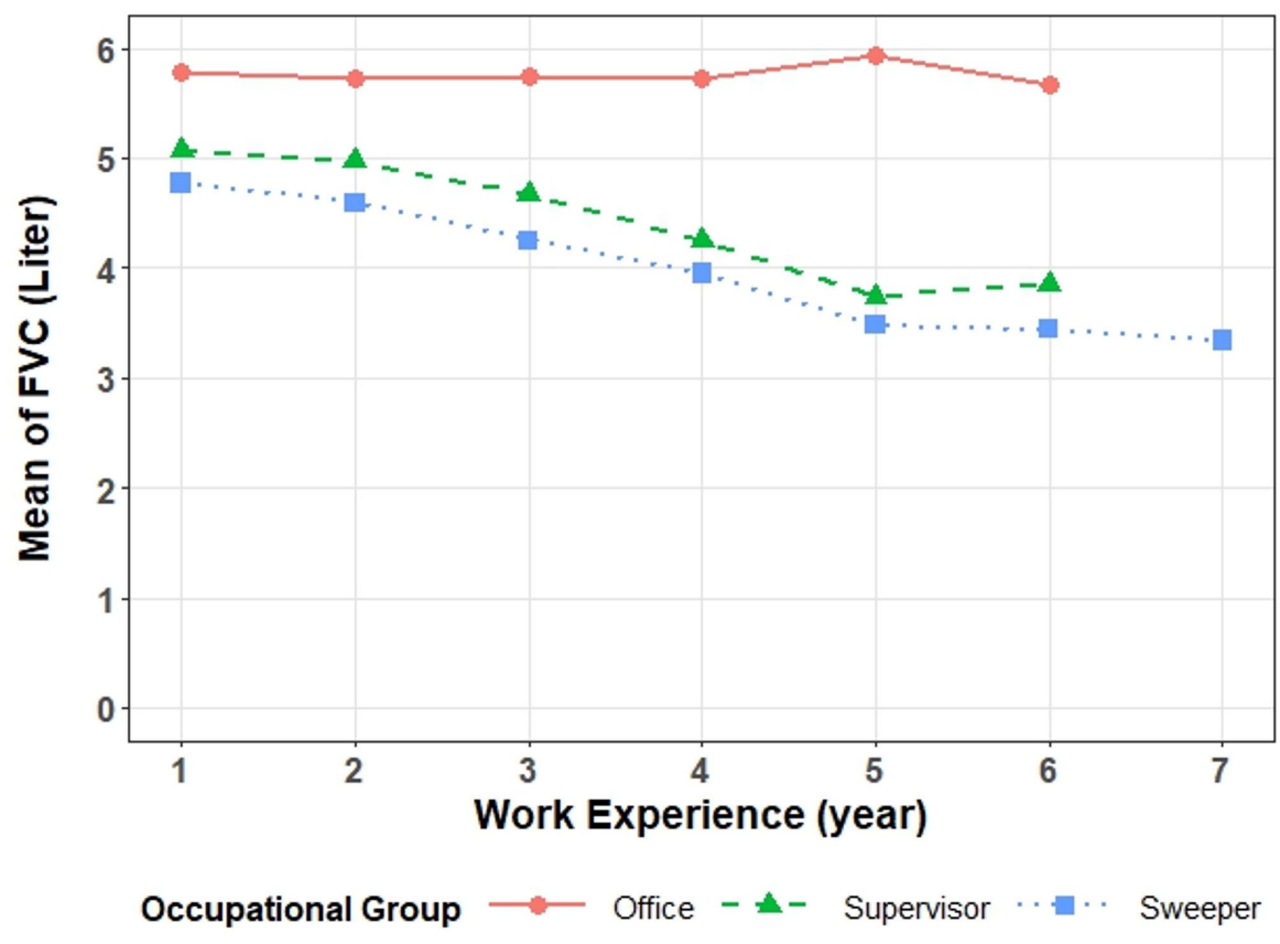
Trend of FVC index changes.

**Fig. 2:**
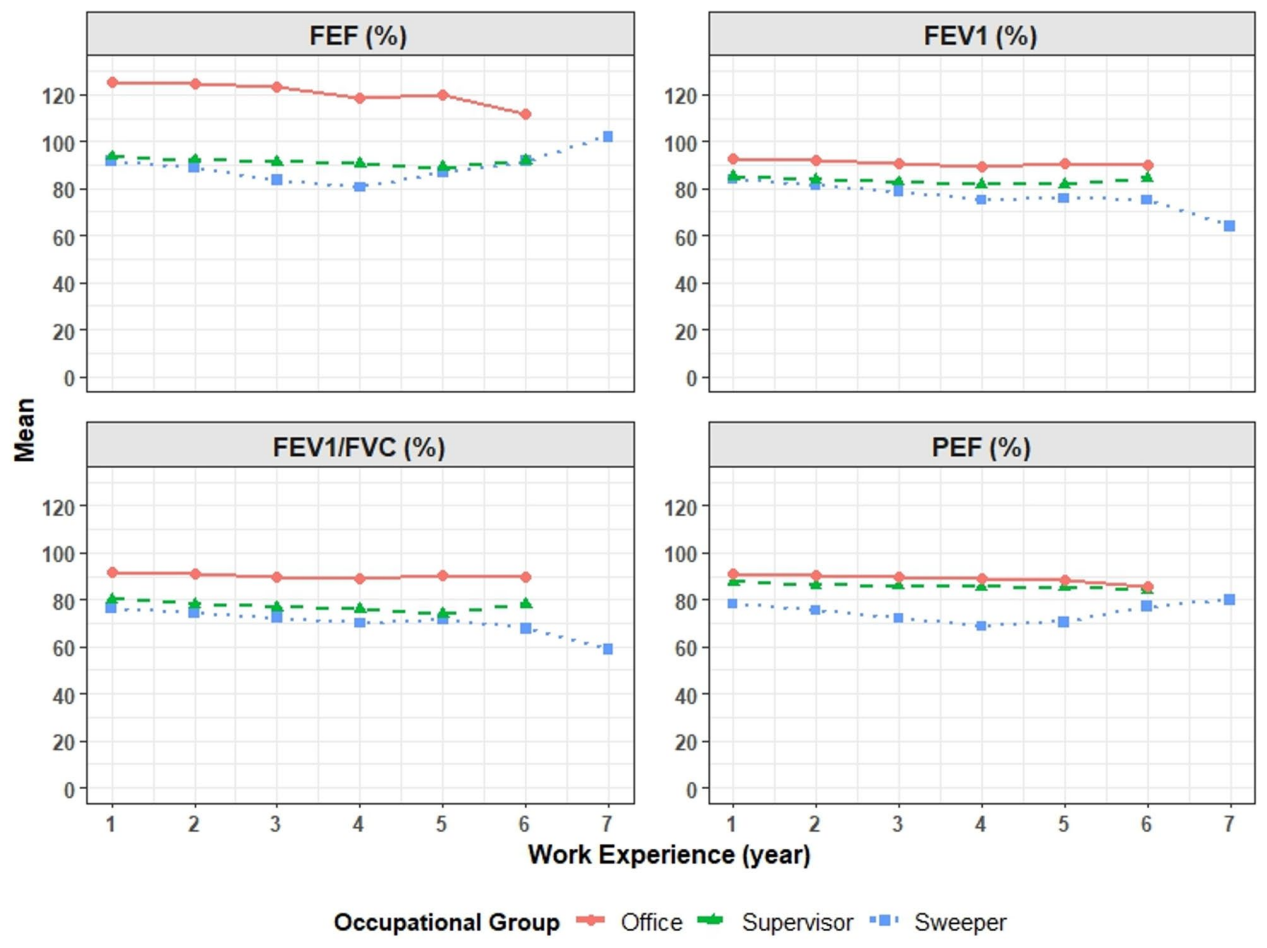
Trend of PEF, FEV_1_, FEF, FEV_1_/FVC index changes.

The longitudinal changes of FVC, FEF, PEF, FEV_1_ and FEV_1_/FVC over time for each occupational group is shown with a diagram (Figs. 1 and 2). In order to investigate the factors affecting this process, two models were conducted and the results are presented by the type of pulmonary function.

### FVC

According to the results of Model 1, while holding work experience constant, the mean FVC index in the sweeper and supervisor groups was significantly lower than in the office group (P-value < 0.001). Furthermore, when the occupational group was held constant, the mean FVC index decreased as work experience increased (P-value < 0.001).

The interaction between occupational group and work experience was also statistically significant (P-value < 0.001), suggesting that the decline rate of the FVC was different between occupational groups, so a more decline was observed in the sweeper and supervisor groups compared to the office group. In Model 2, after adjusting for confounding variables (Cigarette smoking, Waterpipe smoking, Age, Work experience, Type of employment, Type of marriage, and educational level), significant differences were also found in the FVC index of occupational groups. In addition, age (P-value = 0.026), work experience (P-value < 0.001), and education level (P-value < 0.050) were found to be significantly associated with the FVC index.

Even after controlling for other variables (Cigarette smoking, Waterpipe smoking, Type of employment, Type of marriage), the FVC index remained lower in the sweeper and supervisor groups compared to the office group. The interaction between occupational group and work experience remained significant (P-value < 0.001), confirming that the decline in the FVC index was more severe in the sweeper and supervisor groups. Age was positively associated with the FVC index, while work experience had an inverse effect. Individuals with a diploma (P-value = 0.001) or bachelor’s degree (P-value = 0.001) had higher FVC indices than those with a master’s degree or higher. None of the other variables (Cigarette smoking, Waterpipe smoking, Type of employment, Type of marriage) were significantly associated with the FVC index (Table 5).

**Table 5:** Mixed model results for assessing variables associated with pulmonary function.

Model*	Variable	FVC		FEF	
		Regression coefficient (95% CI**)	P-value	Regression coefficient (95% CI**)	P-value
Model 1	Occupational group				
	Office (Ref)	-	-	-	-
	Sweeper group	-0.9 (-0.7, -1.08)	< 0.001	-30.9 (-27.4, -34.4)	< 0.001
	Supervisor group	-0.6 (-0.4, -0.7)	< 0.001	-31.1 (-27.6, -34.6)	< 0.001
	Work experience (year)	-0.06 (-0.04, -0.07)	< 0.001	-0.7 (-0.5, -0.9)	< 0.001
	Interaction between occupational group and work experience				
	Sweeper group*work experience	-0.1 (-0.09, -0.1)	< 0.001	-2.4 (-2.1, -2.6)	< 0.001
	Supervisor group*work experience	-0.07 (-0.05, -0.09)	< 0.001	-0.5 (-0.2, -0.7)	< 0.001
Model 2	Occupational group				
	Office (Ref)				
	Sweeper group	-0.86 (-1.09, -0.64)	< 0.001	-29.18 (-34.02, -24.35)	< 0.001
	Supervisor group	-0.66 (-0.82, -0.49)	< 0.001	-31.36 (-34.84, -27.89)	< 0.001
	Cigarette smoking				
	No (Ref)				
	Yes	-0.11 (-0.26, 0.04)	0.145	-3.66 (-6.86, -0.45)	0.025
	Waterpipe smoking				
	No (Ref)				
	Yes	0.08 (-0.10, 0.27)	0.378	1.78 (-2.23, 5.78)	0.385
	Age	0.01 (0.00, 0.02)	0.026	0.06 (-0.15, 0.28)	0.560
	Work experience (year)	-0.07 (-0.08, -0.05)	< 0.001	-0.81 (-1.10, -0.53)	< 0.001
	Interaction between occupational group and work experience				
	Sweeper group*Work experience	-0.10 (-0.12, -0.09)	< 0.001	-2.40 (-2.67, -2.13)	< 0.001
	Supervisor group*Work experience	-0.07 (-0.09, -0.05)	< 0.001	-0.52 (-0.79, -0.26)	< 0.001
	Body mass index	0.01 (-0.01, 0.03)	0.336	-0.27 (-0.73, 0.20)	0.265
	Type of employment				
	Official recruitment (Ref)				
	Corporate	0.08 (-0.14, 0.30)	0.477	1.99 (-2.72, 6.70)	0.408
	Contractual	0.09 (-0.13, 0.31)	0.439	1.97 (-2.70, 6.65)	0.408
	Type of marriage				
	Single (Ref)				
	Married	-0.05 (-0.20, 0.09)	0.475	2.04 (-1.04, 5.13)	0.195
	Education level				
	Under diploma	0.15 (-0.17, 0.47)	0.368	-6.03 (-12.81, 0.76)	0.082
	Diploma	0.46 (0.18, 0.74)	0.001	5.64 (-0.24, 11.52)	0.060
	Bachelor’s degree	0.42 (0.18, 0.65)	0.001	2.92 (-2.14, 7.98)	0.258
	Master’s degree and above (Ref)				

*Model 1 includes occupational group, work experience, and the interaction between occupational group and work experience. Model 2 incorporates the variables from Model 1, adjusted for cigarette and waterpipe smoking status, type of employment, and marital status. **Confidence Interval.

### FEF

According to the results of Model 1, while holding work experience constant, the mean FEF index in the sweeper and supervisor groups was significantly lower than in the office group (P-value < 0.001). Furthermore, when the occupational group was held constant, the mean FEF index decreased as work experience increased (P-value < 0.001). The interaction between occupational group and work experience was also statistically significant (P-value < 0.001), suggesting that the decline rate of the FEF was different between occupational groups, so a more decline was observed in the sweeper and supervisor groups compared to the office group.

In Model 2, after adjusting for confounding variables (Cigarette smoking, Waterpipe smoking, Age, Work experience, Type of employment, Type of marriage, and educational level), significant differences were also found in the FEF index of occupational groups. In addition, cigarette smoking (P-value = 0.025), and work experience (P-value < 0.001) were found to be significantly associated with the FEF index. Even after controlling for other variables (Waterpipe smoking, Age, Type of employment, Type of marriage, and educational level), the FEF index remained lower in the sweeper and supervisor groups compared to the office group.

The interaction between occupational group and work experience remained significant (P-value < 0.001), confirming that the decline in FEF index was more severe in the sweeper and supervisor groups. work experience and cigarette smoking had an inverse effect. None of the other variables were significantly associated with the FEF index (Table 5).

### PEF

According to the results of Model 1, while holding work experience constant, the mean PEF index in the sweeper and supervisor groups was significantly lower than in the office group (P-value < 0.001). Furthermore, when the occupational group was held constant, the mean PEF index decreased as work experience increased (P-value < 0.001). The interaction between occupational group and work experience was also statistically significant (P-value < 0.001), suggesting that the decline rate of the PEF was different between occupational groups, so a more decline was observed in the sweeper and supervisor groups compared to the office group.

In Model 2, after adjusting for confounding variables (Cigarette smoking, Waterpipe smoking, Age, Work experience, Type of employment, Type of marriage, and educational level), significant differences were also found in the PEF index of occupational groups. In addition, age (P-value = 0.05), and work experience (P-value < 0.001) were found to be significantly associated with the PEF index.

Even after controlling for other variables (Waterpipe smoking, Type of employment, Type of marriage, and educational level), the PEF index remained lower in the sweeper and supervisor groups compared to the office group. The interaction between occupational group and work experience remained significant (P-value < 0.001), confirming that the decline in the PEF index was more severe in the sweeper and supervisor groups. work experience and age had an inverse effect. None of the other variables were significantly associated with the PEF index (Table 6).

**Table 6:** Mixed model results for assessing variables associated with pulmonary function.

Model*	Variable	PEF		FEV_1_		FEV_1_/FVC	
		Regression coefficient (95% CI**)	P-value	Regression coefficient (95% CI**)	P-value	Regression coefficient (95% CI**)	P-value
Model 1	Occupational group						
	Office (Ref)	-	-	-	-	-	-
	Sweeper group	-10 (-8.2, -11.7)	< 0.001	-7.3 (-6.06, -8.5)	< 0.001	-15.1 (-13.6, -16.6)	< 0.001
	Supervisor group	-2.7 (-0.9, -4.4)	0.002	-7.6 (-6.4, -8.9)	< 0.001	-11.6 (-10.1, -13.1)	< 0.001
	Work experience (year)	-0.7 (-0.5, -0.9)	< 0.001	-0.8 (-0.7, -0.9)	< 0.001	-1 (-0.8, -1.1)	< 0.001
	Interaction between occupational group and work experience						
	Sweeper group*work experience	-2.3 (-2.1, -2.4)	< 0.001	-1.7 (-1.5, -1.9)	< 0.001	-0.6 (-0.4, -0.8)	< 0.001
	Supervisor group*work experience	-0.6 (-0.3, -0.8)	< 0.001	-0.3 (-0.1, -0.5)	0.001	-0.4 (-0.3, -0.6)	< 0.001
Model 2	Occupational group						
	Office (Ref)						
	Sweeper group	-8.65 (-10.97, -6.34)	< 0.001	-6.12 (-7.80, -4.43)	< 0.001	-13.66 (-15.77, -11.55)	< 0.001
	Supervisor group	-2.71 (-4.40, -1.02)	0.002	-7.44 (-8.68, -6.21)	< 0.001	-11.66 (-13.19, -10.14)	< 0.001
	Cigarette smoking						
	No (Ref)						
	Yes	-1.01 (-2.52, 0.49)	0.188	-0.49 (-1.58, 0.60)	0.375	-1.18 (-2.57, 0.21)	0.096
	Waterpipe smoking						
	No (Ref)						
	Yes	1.77 (-0.11, 3.65)	0.064	-0.22 (-1.58, 1.14)	0.751	-0.26 (-2.00, 1.47)	0.768
	Age	-0.14 (-0.24, -0.04)	0.005	0.04 (-0.03, 0.11)	0.311	-0.06 (-0.15, 0.03)	0.190
	Work experience (year)	-0.59 (-0.79, -0.38)	< 0.001	-0.88 (-1.04, -0.72)	< 0.001	-0.94 (-1.09, -0.79)	< 0.001
	Interaction between occupational group and work experience						
	Sweeper group*work experience	-2.38 (-2.63, -2.13)	< 0.001	-1.73 (-1.93, -1.52)	< 0.001	-0.65 (-0.82, -0.48)	< 0.001
	Supervisor group*Work experience	-0.61 (-0.85, -0.36)	< 0.001	-0.36 (-0.56, -0.15)	0.001	-0.47 (-0.64, -0.30)	< 0.001
	Body mass index	0.01 (-0.21, 0.23)	0.927	0.00 (-0.16, 0.16)	0.972	0.01 (-0.19, 0.22)	0.889
	Type of employment						
	Official recruitment (Ref)						
	Corporate	0.67 (-1.54, 2.89)	0.551	-2.76 (-4.35, -1.16)	0.001	-0.63 (-2.67, 1.41)	0.544
	Contractual	0.65 (-1.55, 2.84)	0.565	-2.33 (-3.92, -0.75)	0.004	0.23 (-1.79, 2.26)	0.821
	Type of marriage						
	Single (Ref)						
	Married	1.48 (0.03, 2.93)	0.045	0.65 (-0.40, 1.70)	0.224	0.03 (-1.30, 1.37)	0.961
	Education level						
	Under diploma	-4.08 (-7.27, -0.90)	0.012	0.13 (-2.18, 2.43)	0.915	-0.70 (-3.64, 2.24)	0.642
	Diploma	0.13 (-2.63, 2.89)	0.928	2.48 (0.48, 4.47)	0.015	3.63 (1.08, 6.18)	0.005
	Bachelor’s degree	0.79 (-1.58, 3.17)	0.514	2.18 (0.46, 3.90)	0.013	3.06 (0.87, 5.26)	0.006
	Master’s degree and above (Ref)						

*Model 1 includes occupational group, work experience, and the interaction between occupational group and work experience. Model 2 incorporates the variables from Model 1, adjusted for crystalline silica concentration, iron dust concentration, cigarette and waterpipe smoking status, type of employment, and marital status. **Confidence Interval.

### FEV_1_

According to the results of Model 1, while holding work experience constant, the mean FEV_1_ index in the sweeper and supervisor groups was significantly lower than in the office group (P-value < 0.001). Furthermore, when the occupational group was held constant, the mean FEV_1_ index decreased as work experience increased (P-value < 0.001). The interaction between occupational group and work experience was also statistically significant (P-value < 0.001), suggesting that the decline rate of the FEV_1_ was different between occupational groups, so a more decline was observed in the sweeper and supervisor groups compared to the office group.

In Model 2, after adjusting for confounding variables (Cigarette smoking, Waterpipe smoking, Age, Work experience, Type of employment, Type of marriage, and educational level), significant differences were also found in the FEV_1_ index of occupational groups.

In addition, work experience (P-value < 0.001), Type of employment (P-value < 0.050), and Education level except Bachelor’s degree (P-value < 0.05) were found to be significantly associated with the FEV_1_ index. Even after controlling for other variables (Cigarette smoking, Waterpipe smoking, Age, Type of marriage), the FEV_1_ index remained lower in the sweeper and supervisor groups compared to the office group. The interaction between occupational group and work experience remained significant (P-value < 0.001), confirming that the decline in the FEV_1_ index was more severe in the sweeper and supervisor groups. education level were positively associated with the FEV_1_ index, while work experience and type of employment had an inverse effect. None of the other variables (Cigarette smoking, Waterpipe smoking, Age, Type of marriage) were significantly associated with the FEV_1_ index (Table 6).

### FEV_1_/FVC

According to the results of Model 1, while holding work experience constant, the mean FEV_1_/FVC index in the sweeper and supervisor groups was significantly lower than in the office group (P-value < 0.001). Furthermore, when the occupational group was held constant, the mean FEV_1_/FVC index decreased as work experience increased (P-value < 0.001). The interaction between occupational group and work experience was also statistically significant (P-value < 0.001), suggesting that the decline rate of the FEV_1_/FVC was different between occupational groups, so that a more decline was observed in the sweeper and supervisor groups compared to the office group.

In Model 2, after adjusting for confounding variables (Cigarette smoking, Waterpipe smoking, Age, Work experience, Type of employment, Type of marriage, and educational level), significant differences were also found in the FEV_1_/FVC index of occupational groups. In addition, work experience (P-value < 0.001), and education level except under diploma (P-value < 0.05) were found to be significantly associated with the FEV_1_/FVC index. Even after controlling for other variables (Cigarette smoking, Waterpipe smoking, Age, Type of employment, Type of marriage), the FEV_1_/FVC index remained lower in the sweeper and supervisor groups compared to the office group.

The interaction between occupational group and work experience remained significant (P-value < 0.001), further confirming that the decline in the FEV_1_/FVC index was more severe in the sweeper and supervisor groups. education level, except under diploma, were positively associated with the FEV_1_/FVC index, while work experience had an inverse effect. None of the other variables (Cigarette smoking, Waterpipe smoking, Age, Type of employment, Type of marriage) were significantly associated with the FEV_1_/FVC index (Table 6). The changes of each of the pulmonary function indices over time in each studied group are shown in Figs. 1 and 2. It is observed that the values ​​of these indices are the lowest for the sweeper group and the highest for the office group, and the supervisor group is between these two groups.

## Discussion

The present study was conducted in Concentration and Pelletizing plants of an iron ore mining complex. In this study, the workers’ exposure to respirable dust, crystalline silica, and iron dust, as well as their effects on the trend of changes in pulmonary function indices, were investigated. Also, by conducting two statistical models, the effects of parameters such as age, body mass index, marital status, employment status, individual’s level of education, individual’s work experience, and cigarette and waterpipe use on the trend of changes in pulmonary function parameters were measured.

The highest exposure concentration was attributed to sweepers, while the lowest exposure was for office workers. The supervisor group had less exposure than the sweepers and more than the office group. This difference between groups is justified by the type of work people do and the length of time they are exposed to pollutants during a work shift. This means that sweepers spend more time on production lines than supervisors and are therefore exposed to dust for an extended time. On the other hand, the main task of sweepers was manual cleaning of the surfaces with different tools. These tasks cause the dust deposited on the surfaces to rise and spread in the air, increasing the concentration of dust in their respiratory zone.

Furthermore, evidence from other studies aligns with our findings, demonstrating a significant association between occupation and the extent of exposure to environmental pollutants^23^.

These reasons caused the exposure to crystalline silica, respirable dust, and iron dust to be higher than the TLVs for these pollutants in 93.50%, 87.10%, and 19.30% of the surveyed sweepers, while these percentages in the supervisor group were 67.50, 43.20, and 0.00, respectively, and in the administrative group, all exposures were lower than the corresponding TLVs.

In the study by Safinejad et al.^4^ in the Gol Gohar iron ore mine in Sirjan, it was found that the average concentration of the pollutant in the study group was higher than the control group, which was consistent with the results of our study. Additionally, the study by Pourmohammadi et al.^24^ reported that the amount of crystalline silica was in the range of 0.14–1.70 mg/m³, which was higher than that of the control group in the same study. The results of this study also showed that the average concentration of crystalline silica for all people who were exposed to it was higher than the TLVs (0.025 mg/m^3^), which was consistent with our study.

In the present study, the study of the changes in the pulmonary function indices such as FVC, FEV_1_, PEF, FEF and FEV_1_/FVC, over the years of work experience showed that these parameters have a decreasing trend and their average values were lower in the sweepers group than in the supervisors and lower in the supervisors than in the office group. The decreasing trend was more significant in the sweepers than in the supervisors and the office group.

The study by Hashemi Habibabadi et al.^12^showed that exposure to dust in the workplace can reduce pulmonary function parameters in exposed individuals and that pulmonary function parameters, especially PEF and FEF values, were lower in the exposed group than in the control group, which was consistent with the results of this study. Also, a study by Johnsy^13^ yielded similar results, so that the values of FVC, FEV_1_, and FEF were lower in the exposed individuals than in the control group, and the main reason for this was the difference in the duration and concentration of exposure.

Using a linear regression model, factors affecting the trend of each pulmonary function parameter were identified. Factors affecting FVC were occupational group, age, work experience, and education level; for PEF include occupational group, waterpipe smoking, age, and work experience; for FEV_1_, include occupational group, work experience, type of employment, and education; for FEF, include occupational group, smoking, and work experience; and finally, for FEV_1_/FVC were occupational group, work experience, and education level.

In describing the variables that were effective in the trend of pulmonary function parameters in this study, each can be described as follows: with increasing age, the function of body organs becomes weaker, and usually, the respiratory system is no exception to this rule.

Studies conducted in this field also stated that age is related to pulmonary function values, and their relationship is inversely linear^25^. For the variable of work experience, it can also be stated that with increasing years of exposure, the cumulative exposure to the pollutant increases and causes more damage^26^.

Increasing the level of education can lead to improved awareness, attitude, and performance of employees regarding workplace hazards, better effectiveness of training provided to them, and better compliance with work instructions, which can affect their exposure and pulmonary function; studies have also shown that people with lower levels of education do not adequately understand the importance of hazards^27^. The waterpipe and cigarette consumption also affect the pulmonary indices by weakening the respiratory system. Some studies have pointed out that cigarette consumption is one of the most important causes of chronic obstructive pulmonary disease, and also weakens the respiratory system and affects the lung function of individuals^25^.

Regarding the variable of employment type, the monthly income of corporate personnel was lower than that of contractual personnel, and contractual personnel was lower than that of formal personnel, which, according to studies, lower income levels can be associated with worse pulmonary status^28,29^. Gholami et al.‘s study^9^ stated that the pollutant concentration is an influencing factor on the level of pulmonary function parameters which causes them to decrease in the exposed group compared to the control group.

One limitation of this study is that pollutant concentrations were measured only once, which precluded their direct inclusion in the statistical models. Given the longitudinal design, incorporating time-invariant exposure variables (silica and iron concentrations) alongside time-varying outcomes (annual pulmonary function indices) could create temporal inconsistencies and potentially bias the results. Therefore, crystalline silica and iron dust concentrations were excluded from the final model. Instead, job category (sweepers, supervisors, office workers) was used as a proxy for exposure, as these groups differ substantially in expected airborne pollutant exposure. This approach ensured temporal consistency throughout follow-up. Future studies with repeated exposure measurements could address this limitation. Another limitation is that certain potentially influential factors, such as family history of respiratory diseases, past smoking status, and daily consumption among current smokers, were not assessed. Including these variables might have provided a more comprehensive understanding of the study outcomes.

## Conclusions

The exposure of sweepers to respirable dust, iron, and crystalline silica was higher than that of supervisors, while the lowest exposure was observed among office workers. Mean pulmonary function values were lower in sweepers than in supervisors, and highest in office workers. The rate of pulmonary function decline over time was also greater in the sweeper and supervisor groups compared to the office group. These findings underscore the detrimental impact of prolonged exposure to elevated pollutant levels on lung health. In addition to occupational group, factors such as age, work experience, education level, cigarette and waterpipe smoking, and employment type were also significantly associated with pulmonary health. Overall, these results highlight the need for continuous monitoring and effective exposure control measures in occupational settings to protect workers’ respiratory health.

## Supplementary Information

Below is the link to the electronic supplementary material.

Supplementary Material 1

## Data Availability

All data used or analyzed during this study are included in this article and available from the corresponding author upon reasonable request.
