# Supplementary material for: Longitudinal study of pulmonary function trends and associated risk factors in iron ore miners
Source: Sci Rep. 2025 Sep 29;15:33440. doi: 10.1038/s41598-025-19091-7 (PMC12480662; doi:10.1038/s41598-025-19091-7)
Supplement: Supplementary file 1 — Supplementary Material 1 [file 41598_2025_19091_MOESM1_ESM.docx]

**Longitudinal study of pulmonary function trends and associated risk factors in iron ore miners**

Haniyeh Soltanpour ^a^, Ali Faghihi Zarandi ^a^, Abdollah Gholami ^b^, Saiedeh Haji-Maghsoudi ^c^, Behnam Khodarahmi ^d^, Pejman Mohammadi ^e^, Rouhollah Parvari ^f*^

^a^ *Department of Occupational Health Engineering and Safety at Work, Faculty of Public Health, Kerman University of Medical Sciences, Kerman, Iran.*

^b^ *Department of Occupational Health, School of Health, Social Department of Health Research Center, Birjand University of Medical Sciences, Birjand, Iran.*

^c^ *Modeling in Health Research Center, Institute for Futures Studies in Health, Kerman University of Medical Sciences, Kerman, Iran.*

^d^ *Department of Occupational Health Engineering, Gol-E-Gohar Mining and Industrial Co, Sirjan, Iran.*

^e^ *Department of Occupational Health and Safety Engineering, School of Health, Ardabil University of Medical Sciences, Ardabil, Iran.*

^f^ *Environmental Health Engineering Research Center, Kerman University of Medical Sciences, Kerman, Iran*

Figure S1. Calibration curve for crystalline silica standard solution.

Figure S2. Calibration curve for iron standard solution.
